# Relationship between lumbar lordosis and the ratio of the spinous process height to the anterior spinal column height

**DOI:** 10.1038/s41598-020-63648-7

**Published:** 2020-04-21

**Authors:** Hirohiko Inanami, Hiroki Iwai, Takeshi Kaneko, Masahito Oshina, Nodoka Manabe, Yuichi Takano, Yohei Yuzawa, Tomohide Segawa, Kazuyoshi Yanagisawa, Shima Hirai, Fumiko Saiki, Masayoshi Fukushima, Hiroyuki Oka, Ko Matsudaira, Yasushi Oshima, Hisashi Koga

**Affiliations:** 1 Department of Orthopaedic Surgery, Inanami Spine and Joint Hospital, 3-17-5 Higashi-shinagawa Shinagawa-ku, Tokyo, 140-0002 Japan; 2 Department of Orthopaedic Surgery, Iwai Orthopaedic Medical Hospital, 8-17-2 Minamikoiwa Edogawa-ku, Tokyo, 133-0056 Japan; 3 Department of Orthopaedic Surgery, The University of Tokyo, 7-3-1 Hongo, Bunkyo-ku, Tokyo 113-8655 Japan; 4 Department of Medical Research and Management for Musculoskeletal Pain, 22nd Century Medical & Research Center, Faculty of Medicine, The University of Tokyo, 7-3-1 Hongo, Bunkyo-ku, Tokyo 113-8655 Japan

**Keywords:** Musculoskeletal system, Medical research

## Abstract

Purpose Global sagittal imbalance with lumbar hypo-lordosis leads to various problems in elderly populations and is often treated with long-segment fusion and osteotomy. These highly invasive procedures result in a wide range of rigid spines with a high rate of complications. Although some reports have mentioned the primary aetiology of hypo-lordosis, there is limited evidence. Thus, understanding the exact underlying mechanism is required for developing minimally invasive procedures. This study aimed to investigate the factors related to lumbar lordosis (LL) in elderly people. Methods A total of ninety consecutive patients aged ≥ sixty years at a single spine centre were included. We measured LL, the anterior spinal column height consisting of vertebral bodies and intervertebral discs from L1 to L5 (ASC-5) and the sum of the spinous process heights from L1 to L5 (SP-5) with computed tomography in a supine position. The relationship between LL and the SP-5/ASC-5 ratio, SP-5, and ASC-5 was analysed. Results The Pearson correlation coefficients between LL and the SP-5/ASC-5 ratio, SP-5, and ASC-5 were −0.80 (p < 0.001), −0.43 (p < 0.001) and 0.36 (p < 0.001), respectively. Conclusion LL was significantly related to the SP-5/ASC-5 ratio of the lumbar spine in elderly people. In addition to shortening of the ASC, elongation of the SP-5 also contributed to a loss of LL.

## Introduction

Global sagittal spine imbalance with lumbar hypo-lordosis has recently become well known as one of the major causes of reduced health-related quality of life in elderly populations^1,2^. This degenerative condition can lead to various problems including low back pain during standing and walking^2,3^. This outcome is mainly because the back muscles become exhausted to maintain an erect posture while the upper body spontaneously falls forward^2–4^.

Many patients have undergone long-segment spinal fusion surgeries with osteotomy for the correction of sagittal imbalance^5^. These highly invasive operations may cause various perioperative and late complications. Perioperative complications include neurological dysfunction, excessive blood loss, delirium, and infection. Late complications include pseudo-arthrosis, instrumentation and/or graft failure as well as proximal junctional kyphosis^6–8^. Hence, minimally invasive surgical methods are expected to be developed.

To develop such treatments, it is necessary to better understand the aetiology of hypo-lordotic tendencies in the degenerative process. A few reports have stated that weakness and adipose degeneration of the back muscles can be the primary causes^9,10^. Another report mentioned that an increase in spinous process (SP) height may cause hypo-lordosis^11^. Because these studies have demonstrated limited evidence, further investigations are needed to elucidate the underlying mechanism of hypo-lordosis.

Generally, the lack of normal lumbar lordosis (LL) is caused by spinal problems and subsequent age-related degenerative changes^12,13^. Simultaneously, SP height can increase^11,14^, and anterior spinal column (ASC) height can decrease in elderly individuals. These two changes seem to be closely related to hypo-lordosis in the degenerative process. In the present study, we investigated the relationships between these factors and LL in elderly people.

## Materials and Methods

This was a cross-sectional study of data collected from patients with spinal disease.

### Compliance with ethical standards

All procedures were in accordance with the ethical standards of the research committee of Iwai Medical Foundation, and we obtained ethical approval from the committee (No. 20180926-1). Informed consent was obtained from all patients via the disclaimer text on the internet home page of our hospital, according to the law of the Japanese Ministry of Health, Labour & Welfare.

### Patients

A total of 161 consecutive patients who had lumbar computed tomography (CT) images in the supine position at a single spine centre were enrolled for this study from November 1 to November 30, 2018. The exclusion criteria were as follows: age <sixty years, vertebral fracture, history of fusion operation, scoliosis with lumbar curvature with a Cobb angle >15°, spondylolisthesis with Meyerding grade ≥ II, or transitional vertebra due to sacralization or lumbarization. Ninety patients (fifty males and forty females) were included after application of the exclusion criteria. The diagnoses of all patients were reviewed from the electronic medical records. The demographic data of the study population are shown in Table 1, and the diagnoses of the patients are demonstrated in Table 2.

**Table 1:** Demographic information of the study population.

	n	Age (years)		Height (cm)		Weight (kg)		BMI	
		median	IQR	median	IQR	median	IQR	median	IQR
Male	50	71	65.3, 75.0	159.6	150.9, 165.8	61.6	52.4, 65.3	23.4	21.5, 25.2
Female	40	72	68, 76	163.0	155.6, 170.7	66.0	55.7, 69.8	23.2	21.6, 25.5
Total	90	71	67, 75	160.7	153.3, 167.0	62.4	53.8, 69.0	23.3	21.5, 25.4

BMI; body mass index, IQR; interquartile range.

**Table 2:** Diagnosis of the patients.

Disease	Lumbar canal stenosis	Lumbar disc hernia	Listless	Degenerative disc disease	Thoraco-lumbar kyphosis	Baastrup disease
n	65	20	6	5	5	1

Sometimes, a single patient suffered from more than one disease. In these cases, the sum of the number of cases per illness was greater than the actual number of patients (n = 90).

Degenerative disc disease was only diagnosed in patients with symptoms who mainly complained about low back pain. The diagnosis was made according to the criteria reported by Tonosu *et al*.^28^.

### Measurements

A 64-slice CT scanner (Discovery 750 HD/Revolution GSI; GE Healthcare, Tokyo, Japan) was used. The original thickness of the CT images was 0.625 mm, and the re-formatted slice thickness was 2.0 mm. All measurements were performed using imaging software (DICOM Image Work Station XTREK F.E.S.T. A system; J-Mac System Inc., Sapporo, Japan).

### Definitions

**Figure 1:**
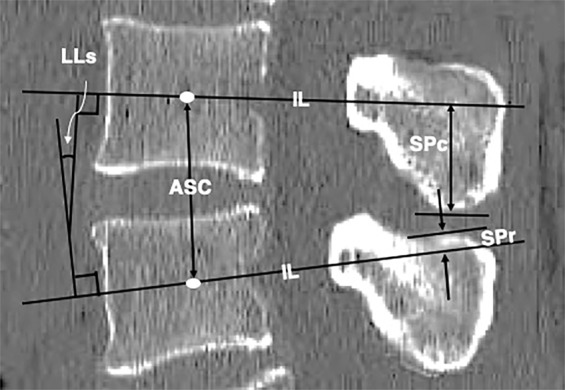
Elements that affect segmental lordosis in a single-motion segment. Segmental lordosis (LLs) is the angle between intersecting lines drawn through the centres of the anterior and posterior walls of the vertebral bodies (IL). The anterior spinal column height (ASC) is the sum of the halves of two relevant vertebral heights and the intervertebral disc height.

For a single-motion segment that included two adjacent vertebrae and one intervertebral disc, we utilized the following four definitions in the mid-sagittal CT image (Fig. 1).

1. Segmental lordosis (LLs): the angle between two intersecting lines drawn through the centres of the anterior and posterior walls (IL) of the upper and lower vertebral bodies.
2. The rostral part of the SP (SPr): the portion of the SP above the IL. The SPr height was defined as the distance between the IL of the lower vertebra and the highest point of the relevant SP.
3. The caudal part of the SP (SPc): the portion of the SP below the IL. The SPc height was defined as the distance between the IL of the upper vertebra and the lowest point of the relevant SP.
4. The ASC height: the distances between the two centres of two adjacent vertebral bodies as the anterior element. This height was calculated as the sum of the halves of two adjacent vertebral heights and the intervertebral disc height.

**Figure 2:**
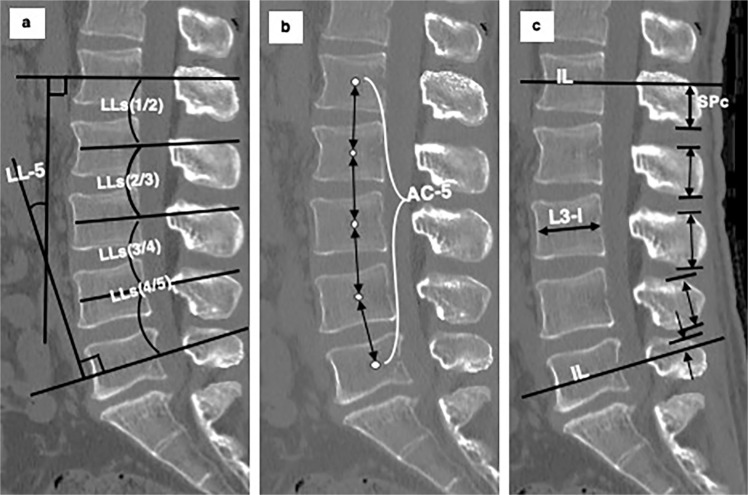
Definitions of L1-L5 parameters in mid-sagittal computed tomography images. (**a**) Lumbar lordosis (LL-5): the angle between intersecting lines drawn through the centres of the anterior and posterior walls of the vertebral bodies (IL) of L1 and L5. LL-5 is the sum of segmental lordosis (LLs) from L1/2 through L4/5. (**b**) Sum of the anterior spinal column height (ASC-5): the sum of the distances between the centres (white dots) of the adjacent vertebral bodies of L1-L2 through L4-L5. (**c**) Sum of spinous process heights (SP-5): the L1 SPc height (the height of the caudal part of the spinous process) + whole SP heights of L2, L3 and L4+ L5 SPr height (the height of the rostral part of the spinous process).

These four definitions made it possible to calculate the parameters of the whole lumbar spine by adding the measured values of each motion segment together. LL-5 represents the sum of LLs from L1/2 through L4/5 (Fig. 2a). ASC-5 represents the sum of ASC heights from L1–L2 through L4–L5 (Fig. 2b). SP-5 represents the sum of SPc of the L1 SP, the whole SP height of L2, L3, and L4 and the SPr of the L5 SP (Fig. 2c).

### Normalization

To control the variance in individual body habitus, the computed values of ASC-5 and SP-5 were divided by the relevant L3 vertebral body antero-posterior width, which tends to be maintained in elderly people^15^ (normalized ASC-5 and normalized LL-5).

### Statistical analysis

We performed statistical analysis using SPSS 24.0 (SPSS Inc., Chicago IL, USA). The relationships between LL-5 and the following three parameters were separately assessed using the Pearson correlation coefficient: the 1) SP-5/ASC-5 ratio, 2) normalized SP-5 and 3) normalized ASC-5.

### Measurement reliabilities

The intra-class correlation coefficient (ICC) was assessed in terms of these four parameters for intra-rater reliability. We defined ICC values less than 0.5 as indicative of poor reliability, values between 0.5 and 0.75 as indicative of moderate reliability, values between 0.75 and 0.9 as indicative of good reliability, and values greater than 0.90 as indicative of excellent reliability. All reported p values were two-tailed, with differences reported as significant when p < 0.05.

A single observer measured all four parameters (LL-5, SP-5, ASC-5 and the L3 vertebral body antero-posterior width) of the 27 patients (12 females and 15 males), representing 30% of the study participants, twice with at least a one-week interval to assess the ICC.

## Results

The ICC values, 95% confidence intervals, and p-values of LL-5, SP-5, ASC-5 and the L3 vertebral body antero-posterior width were 0.911 (0.814–0.958; p < 0.0001), 0.978 (0.952–0.990; p < 0.0001), 0.971 (0.937–0.986; p < 0.0001), and 0.938 (0.869–0.971; p < 0.0001), respectively. All results indicated excellent intra-rater reliability.

Table 3 shows the median and interquartile range of these four parameters.

**Table 3:** Median and IQR of the measured parameters.

	n	LL		SP-5		ASC-5		L3-l		SP-5/ASC-5	
		median	IQR	median	IQR	median	IQR	median	IQR	median	IQR
Male	50	21.6	15.7–28.0	104	99.6–112.5	137.3	130.3–143.8	33.5	32.4–34.7	0.78	0.72–0.82
Female	40	21.3	14.4–30.8	96.4	91.7–103.9	129	124.3–131.8	30.6	28.2–32.3	0.75	0.72–0.80
Total	90	21.6	15.0–29.5	100.9	95.4 109.2	131.8	126.8 140.4	32.6	30.6 33.9	0.77	0.72–0.81

SP; spinous process, ASC; anterior spinal column, L3-l; L3 vertebral body antero-posterior width, IQR; interquartile range.

**Figure 3:**
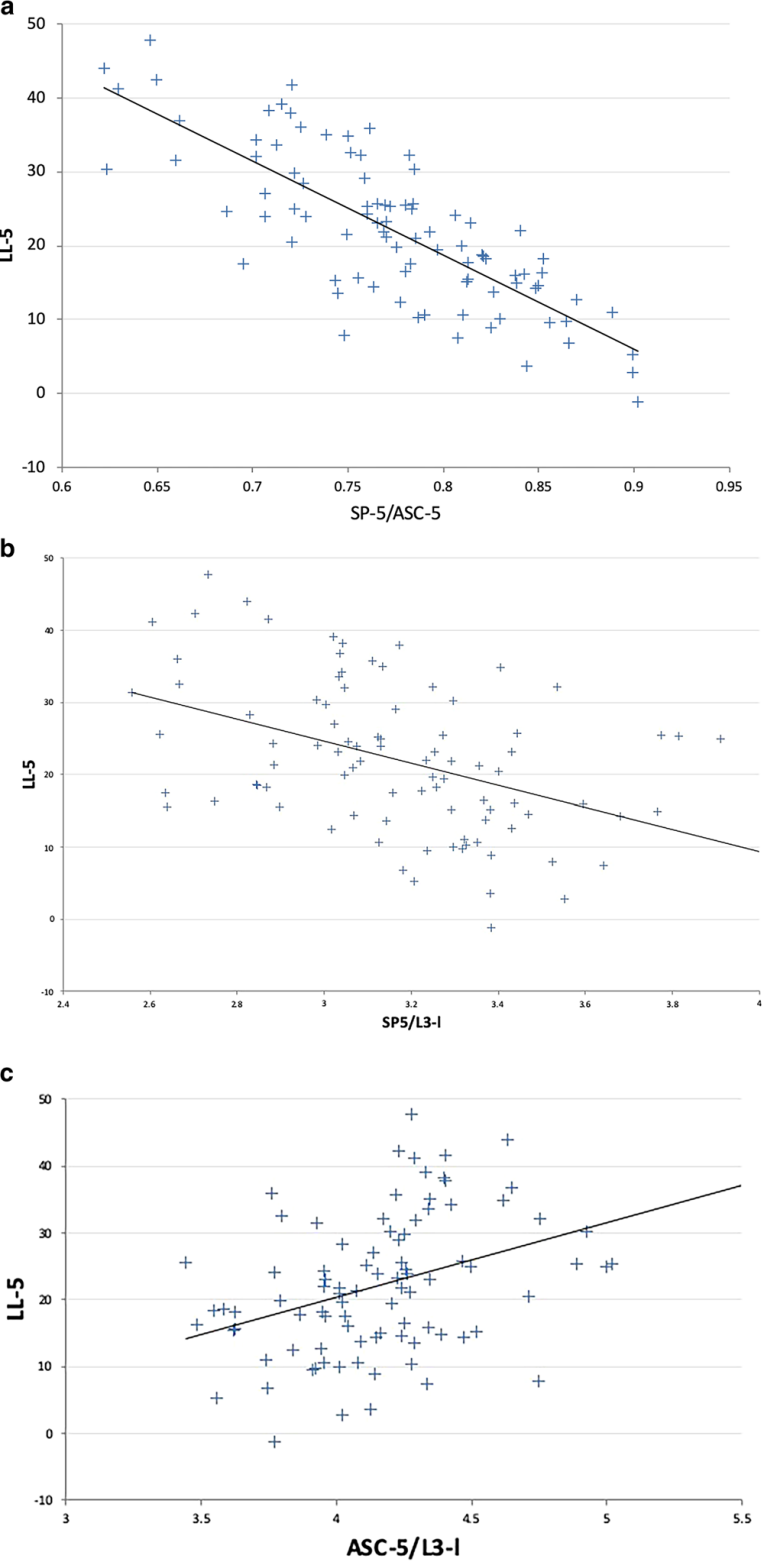
Scatter plot of the relationship between LL-5 and the three parameters. (**a**) LL-5 and the SP-5/ASC-5 ratio, R = −0.80 (p < 0.001). (**b**) LL-5 and normalized SP-5 (SP-5/L3-l), R = −0.43 (p < 0.001). (**c**) LL-5 and normalized ASC-5 (ASC-5/L3-l), R = 0.36 (p < 0.001). LL-5: the angle between intersecting lines drawn through the centres of the anterior and posterior walls of the vertebral bodies of L1 and L5. SP-5: the height of the caudal part of the L1 spinous process + whole spinous process heights of L2, L3 and L4+ the height of the rostral part of the L5 spinous process. ASC-5; the sum of the distances between the centres of the adjacent vertebral bodies of L1-L2 through L4-L5. L3-l; L3 vertebral body antero-posterior width.

The Pearson correlation coefficient between LL-5 and the SP-5/ASC-5 ratio was −0.80 (p < 0.001). On the other hand, the coefficients between LL-5 and normalized SP-5 and between LL-5 and normalized ASC-5 were −0.43 (p < 0.001) and 0.36 (p < 0.001), respectively. A scatter plot of the relationship between LL-5 and these three parameters is shown in Fig. 3.

**Figure 4:**
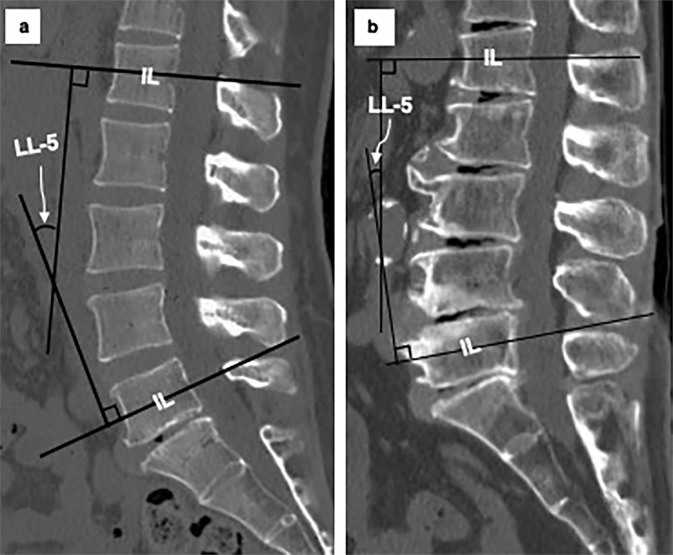
Typical cases of normal lordosis and hypo-lordosis. (**a**) Case of normal lordosis: the height of the anterior spinal column is maintained, and the spinous process height is limited in the posterior elements, allowing lordosis of the lumbar spine. (**b**) Case of hypo-lordosis: the length of the anterior spinal column is shortened, and the spinous process height is elongated. The remodelling or hypertrophy of the spinous processes is observed mainly at the attached portion of the thin and weak interspinous ligament, not at the strong supraspinous ligament, especially at the L2/3, L3/4, L4/5 and L5/S1 levels.

Figure 4 shows two typical cases of normal lordosis (a) and hypo-lordosis (b). The height of the ASC is maintained in the case of normal lordosis but is shortened in the case of hypo-lordosis. In addition, the height of the SP is elongated at every spinal segment in the case of hypo-lordosis compared to the case of normal lordosis.

## Discussion

Complicated and highly invasive surgical procedures have been performed to correct global sagittal imbalance^5^. Global sagittal imbalance is affected by many factors, such as LL, thoracic kyphosis, pelvic incidence, pelvic tilt, and sacral slope. Among them, LL and pelvic incidence are so important that many surgeons set surgical goals by determining the ideal degree of LL as a target value based on individual pelvic incidence^16^. However, to develop minimally invasive treatment methods, it is necessary to elucidate the detailed aetiology of the decreasing tendencies of LL. Only a few reports have mentioned that weakness and/or adipose degeneration of back muscles are primary causes with limited evidence^9,10^, but the factors of lumbar spinal morphology that contribute to the development of kyphosis have not been well studied.

The lumbar spine of a newborn is kyphotic^17^. Within three years after birth, a human acquires LL along with an erect posture and bipedal locomotion^17^. The early increase in lumbar lordosis relies on the development of segmental inclination, which is gradually replaced by remodelling of the intervertebral disc shape and, later in life (adult period), by remodelling of the vertebral bodies^18^. It has also been reported that anterior spine elements develop faster than posterior counterparts, which results in normal LL during the growth period^19^. Lordosis gradually increases until the age of approximately 20 years old^17,20^, and the curve is maintained for a while^21^. Generally, the loss of normal LL is caused by age-related degenerative changes^12,13^. There are some reports regarding the decrease in LL in elderly populations. One paper mentioned that LL tended to decrease after 60 years of age^22^, and another paper stated that LL decreased after forty to fifty years of age^23^.

LL is determined by the relationship between the height of anterior elements and that of posterior elements. The longer the posterior elements are in relation to the anterior elements, the smaller LLs becomes. At a single-motion segment, the anterior elements consist of halves of the relevant two vertebrae and the intervertebral disc, whereas the posterior elements include the caudal portion of the upper SP (SPc), the rostral portion of the lower SP (SPr) and the length of the interspinous ligament (Fig. 1). Therefore, both ASC height loss due to degenerative disc space narrowing or vertebral compression and SP height increases can contribute to the development of lordosis loss, i.e., kyphosis.

Disc space narrowing is a well-known degenerative change in the spinal axis, but an increase in the SP height of posterior elements has also been observed as one of the degenerative changes^11,14^. An inverse relationship between LL and SP height has been previously reported^11^, as we showed in the present study. Two causality hypotheses have been proposed: a) the decrease in LL causes elongation of the SP, and b) elongation of the SP causes a decrease in LL. In the former hypothesis, the disc height first decreases with progressive degeneration, while the posterior side of the spine is anchored by the facet joint, which leads to a decrease in lordosis. Kyphotic angulation increases the tension of the interspinous and supraspinous ligaments, which in turn triggers an adaptive response within the SP and results in an increase in its height. This scenario seems compatible with Wolff’s law, which explains the reactive changes in bone morphology, but it is of note that the remodelling or hypertrophy of the SP is mainly observed not at the location where the strong supraspinous ligaments attach but where the thin and membranous interspinous ligaments^24^ attach. The latter scenario is that enlarged SPs contribute to a decrease in LL. Although the true mechanism of the remodelling of SPs is unknown, the enlargement of SPs blocks the shortening of posterior elements on extension and thus has a kyphosing effect per se. As we found a significant correlation between LL-5 and SP-5 in the present study, we speculate that SP enlargement also contributes to the development of LL loss independently of ASC height decreases.

Table 4 summarizes previous reports that discussed SP height and its impact on other spinal parameters. It is worth noting that Aylott *et al*. recently focused on the significance of SP height and its relation to LL and reported a reverse relationship between these two parameters^11^. Nevertheless, they did not show correlation coefficient data and did not consider anterior elements such as the ASC. Another report^14^ discussed the ASC and SP height but not LL. According to the present study, the correlation coefficient between LL and SP height was similar to that between LL and ASC (−0.43 and 0.36, respectively). This result demonstrates that both anterior and posterior elements play equally important roles in determining LL in elderly populations.

**Table 4:** Comparison with other reports.

	Year	Measurement of LL	Measurement of SP	Measurement of ASC	Analysis method
Aylott C E W *et al*.^11^	2011	L1/S1 (upper endplates)	L1 - L5	no	Linear regression models
Paholpak P *et al*.^14^	2013	no	L4, L5	L4, L5	Welch t test
Present study		L1/5 (between each intersecting line)	L1 - L5	L1 - L5	Pearson correlation coefficient

SP; spinous process, ASC; anterior spinal column.

Aylott *et al*.^11^ reported that the rate of increase in SP height was 0.03–0.07 mm/year and that lordosis decreased by 1° on average for each additional millimetre in average height.

We believe that understanding the concept of the SP-5/ASC-5 ratio is useful for determining surgical strategies to correct decreased LL in patients with sagittal imbalance. Highly invasive long-segment fusion surgery with posterior wedge osteotomy such as pedicle subtraction osteotomy^25^ is still the mainstream for these cases. The essence of these operations is the shortening of the posterior elements^5^. However, in cases of high SP-5/ASC-5 ratios, where the size of the SP has a significant contribution, less invasive treatment methods can be feasible via partial resection of the SP while obtaining elongation of the anterior elements via extension exercises and/or anterior interbody fusion techniques.

This study has some limitations that should be considered. First, the impact of the SP-5/ASC-5 ratio on LL may be different based on the extent of spinal degeneration (i.e., narrowing disc space and elongating SP height). Further investigations involving a larger and more diverse study population are needed to determine the detailed effect of each factor. Second, the image data were acquired in the supine position. LL should be measured when the patient is in a standing position to consider global alignments^12^. Conventional X-ray devices can provide standing images of the spine but do not have enough resolution to precisely gauge SP height. Many reports have shown that the degree of LL is smaller in the supine position than in a standing position^26^. However, one report showed that the degree of LL in adult-to-elderly patients with spinal deformities was found to be larger in the supine position than in a standing position^27^. Hence, the evaluation of LL by CT or magnetic resonance imaging should be considered in combination with the assessment of LL in a standing position. Third, we did not include a healthy control group in the analysis because it was difficult to obtain CT images in a healthy control group. Fourth, the data were collected from only one facility. Finally, the study population included only elderly Asian individuals; therefore, the results may not be generalizable to other demographic groups.

## Conclusion

LL was significantly related to the SP-5/ASC-5 ratio of the lumbar spine in elderly people. In addition to shortening of the ASC, elongation of the SP-5 also contributed to a loss of LL.

## Data Availability

The datasets generated and analysed during the current study are available from the corresponding author upon reasonable request.
